# Deep Learning-Enabled Flexible PVA/CNPs Hydrogel Film Sensor for Abdominal Respiration Monitoring

**DOI:** 10.3390/gels11090743

**Published:** 2025-09-16

**Authors:** Chengcheng Peng, Xinjiang Zhang, Ziyan Shu, Cailiu Yin, Baorong Liu

**Affiliations:** 1 Guangxi Colleges and Universities Key Laboratory of Environmental-Friendly Materials and New Technology for Carbon Neutralization, Guangxi Key Laboratory of Advanced Structural Materials and Carbon Neutralization, School of Materials and Environment, Guangxi Minzu University, Nanning 530105, China; 2 School of Physical Science & Technology, Guangxi University, Nanning 530004, China

**Keywords:** hydrogel film, multi-functional, deep learning, 1D-CNN, sandpaper templates

## Abstract

In this study, a flexible hydrogel film sensor based on the intermixing of poly(vinyl alcohol) (PVA) and biomass-derived carbon nanoparticles (CNPs) was prepared and microstructures were constructed by replicating sandpaper templates on its surface. The sensor thus has good overall sensing performance with a sensitivity of 101 kPa^−1^, a fast response/recovery time of 22 ms and 20,000 fatigue cycles. The sensor was experimentally verified to accurately capture human joint movements, current signals of written letters, and weight differences in the size of spherical objects. Based on this, a breathing phase classification framework was constructed using the 1D-CNN algorithm, achieving a synergistic enhancement effect between environmentally scalable materials and Deep learning algorithms. This approach not only improves the signal discrimination function, but also provides new ideas for wearable medical monitoring, haptic feedback and intelligent robot human–machine interface.

## 1. Introduction

Flexible pressure stimulated sensors have been widely used in cutting-edge fields such as wearable devices [1], soft robotics [2], human–computer interaction [3] and healthcare [4,5] due to their advantages of convenient signal acquisition and simple preparation process. Thanks to their excellent flexibility, high sensitivity and fast response time, these sensors show great promise for real-time monitoring of human physiological parameters. However, the core challenge of the current research is to develop flexible sensor devices with high electrical conductivity, excellent mechanical stability and long-term reliability, especially smart sensor systems that can accurately capture the human body’s movement state and monitor weak physiological signals in real time.

Flexible pressure sensors can be classified into four main types based on their operating principles: piezoelectric, piezoresistive, capacitive, and triboelectric sensors [6]. Among these sensing mechanisms, piezoresistive sensors achieve pressure detection by converting external pressure signals into resistance or current changes. These sensors are of interest because of their simple structural design, lower manufacturing cost, and high detection sensitivity [7]. The material system of flexible piezoresistive sensors principally comprises two categories: namely, conductive materials and matrix materials. In recent years, research on conductive materials has focused on carbon-based nanomaterials (e.g., carbon nanotubes [8,9] and graphene [10,11]), metallic nanomaterials [12,13], and two-dimensional transition metal carbon-nitrogen compounds (MXene) [14]. However, these materials generally suffer from complex preparation processes and high production costs. In this context, the development of low-cost and easy-to-prepare novel conductive fillers has become a research hotspot. Among them, biomass carbon materials show good application prospects in the construction of piezoresistive flexible pressure sensors due to their advantages of wide sources and environmental friendliness. Currently, studies have been conducted to successfully prepare piezoresistive flexible pressure sensors with excellent performance by using various biomass raw materials as conductive fillers, such as cotton [15], corn stalks [16] and peanut shells [17]. However, these traditional biomass carbon materials often suffer from poor dispersion and unstable conductive network construction due to their irregular morphology, which seriously limits the improvement of sensor performance. Given these limitations, the study of biomass carbon in piezoresistive sensors is still in its infancy and there is an urgent need to develop novel structural materials. In this context, cuttlefish ink (CI) is a promising biomass carbon material because it naturally contains monodisperse spherical particles (100–150 nm). Carbonization of these particles yields conductive carbon nanospheres (CNPs) that overcome the morphological limitations of conventional biomass materials. When CNPs are introduced into the design of piezoresistive sensors, a polymer matrix capping strategy is typically employed. Polyvinyl alcohol (PVA) is particularly advantageous as a vinyl polymer matrix due to its unique physical properties, including abundant hydroxyl groups, strong hydrogen bonding capacity, high dielectric strength, excellent charge storage capability, and remarkable optical properties [18,19,20]. In this context, solution casting of PVA-based polymer composites emerges as an ideal fabrication approach, offering a simple, cost-effective, and scalable method for producing thin-film piezoresistive sensors [21,22]. However, due to the lack of customised surface morphology, conventional planar thin films often suffer from low response sensitivity, significant signal hysteresis, complex fabrication processes and delayed response to physical stimuli [23]. In order to address these problems and integrate microstructures in wearable pressure sensors, various surface engineering methods have been employed, including photolithography [24], templating [25] and 3D printing [26]. Pressure sensors based on directly templated silk and leaf [25] microstructures have been reported to have high sensitivity [27]. It is worth noting that among the many templating materials, sandpaper exhibits significant advantages due to its unique surface properties its high surface roughness and uniform roughness distribution, a feature that makes it an ideal mould material for generating high-performance microstructured sensors.

Although microstructures can improve sensitivity, they still face technical bottlenecks in scenarios such as respiratory phase capture, which requires the collaborative optimisation of material-device-algorithm systems and the development of deep-learning models adapted to dynamic physiological signals (e.g., abdominal respiration), in order to break through the limitations of traditional signal processing methods. Traditional respiration monitoring techniques rely on bulky hardware or complex manual calibration, which are not sensitive enough and are susceptible to environmental disturbances, leading to limited dynamic human feature extraction [28,29,30]. Therefore, the development of scalable automated classification algorithms has become crucial, and AI techniques provide the means to do so by efficiently parsing sensor data. Deep learning as a subset of AI automatically extracts intrinsically relevant features from data and has shown benefits in areas such as image recognition and behavioural monitoring [31,32,33,34]. Its end-to-end learning approach effectively reduces the need for manual feature extraction engineering and has relatively low computational loss compared to more complex computational models while maintaining high accuracy. When dealing with time-series sensor data [35], one-dimensional convolutional neural networks (1D-CNN) show excellent performance in effectively modelling local temporal dependencies and extracting multi-level temporal features by stacking convolutional layers. Enabling 1D-CNN to directly process raw signals, they are ideal for wearable sensor applications and human activity detection [36].

In recent years, Wang’s team [37] has developed a flexible pressure sensor system for monitoring infant respiration, utilising integrated circuit design to create a sensor that is fixed to the infant’s waist to enable real-time data collection. The sensitivity of this device is approximately 30.7 kPa^−1^. However, such devices still have limitations in terms of sensitivity, response speed, and lack of intelligent data processing. In contrast, this study developed a sandpaper microstructure PVA/CNPs hydrogel film sensor with a sensitivity of 101 kPa^−1^ and a response/recovery time of only 22 milliseconds. However, it can also be quickly integrated with 1D-CNN to capture high-quality respiratory signals, achieving classification accuracies of 90.6% and 94.8% during exhalation and inhalation phases, respectively. This fully demonstrates the immense potential of combining piezoresistive sensors with AI.

## 2. Results and Discussion

### 2.1. Structure Characterisation

**Figure 1:**
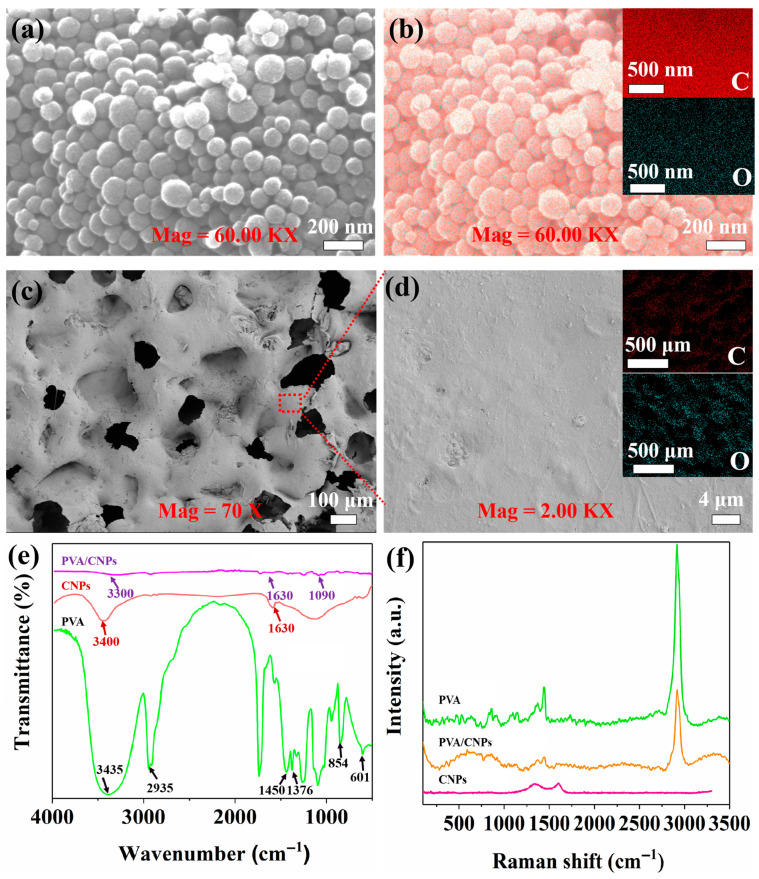
(**a**) SEM micrograph of CNPs. (**b**) Surface scan of CNPs with inset EDS spectra showing carbon C and oxygen O distribution. (**c**) SEM image of the surface of PVA/CNPs hydrogel film sensor replicating the microstructure of sandpaper. (**d**) Enlarged view of the blue dashed area in (**c**). The inset is an EDS spectrum showing the elemental distribution of C and O in the whole region shown in (**c**). (**e**) FTIR spectra of PVA, CNPs and PVA/CNPs composites. (**f**) Raman spectra of PVA, CNPs and PVA/CNPs composites.

The high sensitivity performance of PVA/CNPs hydrogel film sensor mainly stems from their microstructural features. For this reason, the morphology of the PVA/CNPs composite hydrogel film with sandpaper-like surface structure was analysed by SEM. As shown in Figure 1, the microstructures of the composites and their elemental distributions were systematically characterised by combining SEM and energy spectroscopy (EDS) techniques. The SEM micrograph of CNPs in Figure 1a shows a uniformly distributed spherical structure with an average particle size of about 100 nm. Figure 1b provides the EDS spectra of the CNPs, confirming the presence of carbon (C) and oxygen (O) as major elements. This uniform elemental distribution highlights the successful preparation of CNPs. Figure 1c illustrates the SEM image of the surface of the PVA/CNPs hydrogel film sensor, which shows the porous microstructure. Interestingly, the surface morphology of the film successfully replicates the microstructure of the sandpaper used in the fabrication process, as the PVA/CNPs hydrogel film was peeled off from the sandpaper. The successful replication of the reverse RHD spinosum microstructure was confirmed by the formation of irregular pores [38] in the PVA/CNPs hydrogel film sensor caused by the depression of the substrate by the RHD diamond grit on the sandpaper during the curing process. This structure and its surface grooves and recesses create space for deformation [39]. Often this structure plays a more important role in improving the response speed and increasing the pressure detection range of piezoresistive sensors. Figure 1d is an enlarged view of the blue dashed area in Figure 1c, where one can observe the presence of flat areas in addition to the surface groove or notch structure. The accompanying EDS spectra in Figure 1d show the overall distribution of elements in the region shown in Figure 1c, further verifying the uniform distribution of carbon (C) and oxygen (O) elements. This observation illustrates the effective binding of CNPs to PVA polymers.

The FT-IR spectra in Figure 1e highlight the structural and interactional features of PVA, CNPs, and PVA/CNPs composites, showing notable differences. The PVA spectrum shows a broad band at 3435 cm^−1^ for O–H stretching vibrations from both PVA and water molecules. The 2935 cm^−1^ peak corresponds to C–H stretching vibrations [40], with C–H and O–H bending at 1450 cm^−1^, and a weak CH_2_ wagging at 1376 cm^−1^. Peaks at 854 cm^−1^ and 601 cm^−1^ are associated with C=C stretching and weak O–H wagging vibrations, respectively. In contrast, the CNPs spectrum displays minimal peaks, with a weak band at ~1630 cm^−1^ that could possibly be attributed to C=O stretching vibrations from surface-bound functional groups or adsorbed water. A faint O–H stretching signal near ~3400 cm^−1^ may suggest the presence of limited hydroxyl groups. Upon forming PVA/CNPs composites, the O–H stretching band of PVA is significantly reduced in intensity, which could indicate possible disruptions to the hydrogen-bonding network due to interactions with CNPs. The band at ~1630 cm^−1^ becomes more prominent, likely resulting from contributions of the CNPs surface groups. Furthermore, subtle shifts in the C–O/C–O–C stretching region (~1090 cm^−1^) may suggest strong molecular interactions between PVA and CNPs.

The Raman spectra, as shown in Figure 1f, reveal distinct structural features of the components. For CNPs, the characteristic peaks at ~1345 cm^−1^ (D-band) and ~1588 cm^−1^ (G-band) correspond to disordered carbon structures and graphitic carbon, respectively. The relative intensity ratio of the (D band) to the (G band) of CNPs is 0.499 [41]. A higher *I*_D_/*I*_G_ ratio typically indicates a lower degree of disorder and a higher degree of graphitisation in carbon materials. In the PVA spectrum, a peak around 3000 cm^−1^ is linked to O–H stretching vibrations, while the 500–1500 cm^−1^ range corresponds to C–C and C–H vibrations typical of the polymer backbone [42]. The PVA/CNPs spectrum combines features of both components, with the D-band and G-band of CNPs remaining prominent and the O–H peak of PVA exhibiting reduced intensity, indicating possible hydrogen bonding or molecular interactions between PVA and CNPs.

### 2.2. Sensing Properties

**Figure 2:**
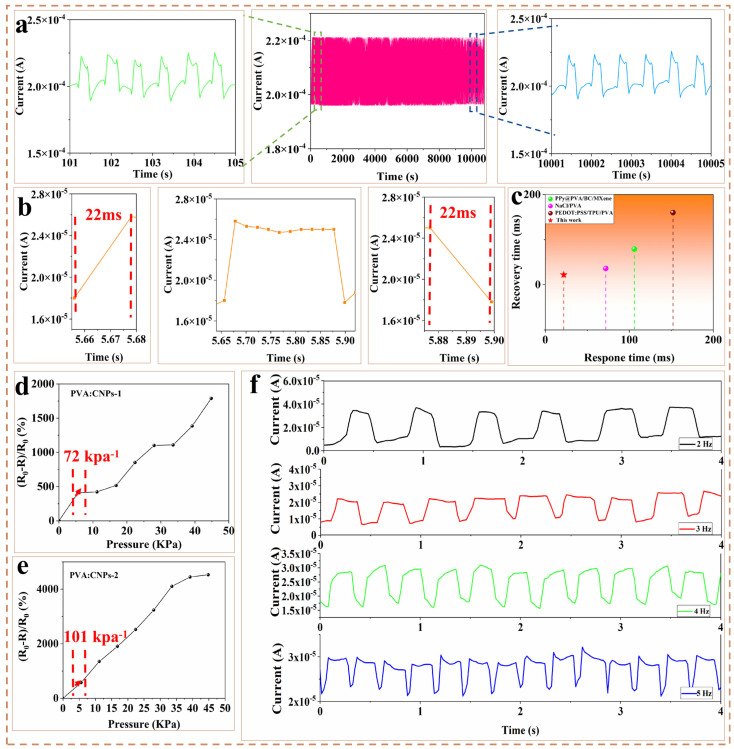
Sensing performance of PVA/CNPs hydrogel film sensor. (**a**) Fatigue test of PVA/CNPs hydrogel film sensor at 2 Hz for 20,000 cycles. (**b**) Response time and recovery time of PVA/CNPs hydrogel film sensor. (**c**) Comparison of this study (PVA/CNPs-2) with previously reported studies [45,46,47] in terms of response time and recovery time. (**d**) Sensitivity of PVA/CNPs-1 and (**e**) PVA/CNPs-2 in the 0–44.8 kPa range. (**f**) Sensing performance of the sensors at 2 Hz, 3 Hz, 4 Hz and 5 Hz, respectively.

PVA/CNPs hydrogel film sensor are suitable for pressure sensors due to their good electrical conductivity and excellent fatigue resistance. To assess the stability and lifetime of the PVA/CNPs sensor film over a long period of time, a compression test of 20,000 cycles at 1 Kpa pressure and 2 Hz frequency was performed (Figure 2a). The stable current response indicates that the sensor has good fatigue resistance. The PVA/CNPs thin film sensors exhibited excellent signal response (Figure 2b) with a response time of 22 ms during loading and unloading and a recovery time of 22 ms, making them suitable for real-time monitoring in practical applications. Compared with previously reported PVA-based sensors with different preparation methods and materials (Figure 2c), the PVA/CNPs hydrogel film sensor offer significant advantages in terms of response time and recovery time.

To evaluate the sensor’s sensitivity, the sensitivity parameter is measured in this paper, and the sensitivity (S) is calculated according to the following equation:

$$S=\frac{(R_{0}-R)/R_{0}}{\Delta P}$$

where (*R*_0_ − *R*)/*R*_0_ is the relative resistance change, and (Δ*P*) is the pressure change.

Figure 2d,e show the sensitivity curves of PVA/CNPs-1 and PVA/CNPs-2. In the pressure range of 0–5.6 kPa, the sensitivity of PVA/CNPs-1 is 72 kPa^−1^, while the sensitivity of PVA/CNPs-2 is clearly higher at 101 kPa^−1^ The significant increase in the sensitivity can be attributed to the higher content of CNPs in PVA/CNPs-2 compared to PVA/CNPs-1. The higher CNPs content improves the density and connectivity of the conductive network [43], allowing the CNPs to interconnect more efficiently under applied pressure. This enhanced interconnectivity amplifies the variation in electrical resistance during compression, resulting in the superior sensitivity of PVA/CNPs-2. Figure 2f shows the test results of the current variation in the sensor at different frequencies, specifically 2 Hz, 3 Hz, 4 Hz and 5 Hz. At 2 Hz, the current waveform is relatively smooth and fluctuates within a certain range. The peaks and troughs are not very sharp and the spacing between each waveform is relatively large. Compared to the waveform at 2 Hz, the oscillations are more frequent at 3 Hz due to the increased frequency. The current value between the peaks and troughs changes more rapidly. When the frequency is increased to 4 Hz, the current waveform becomes finer and the fluctuations occur more quickly. Finally, at a frequency of 5 Hz, the current shows the most rapid oscillations. Each waveform is closely spaced. This performance at different frequencies demonstrates the sensor’s ability to adapt to sensing applications under different speed conditions. Table 1 compares the pressure detection performance of the proposed sensor with that of recently reported sensors under room temperature and ambient humidity conditions. The test results show that the sensor exhibits high sensitivity (101 kPa^−1^), a detection range (0–44.8 kPa), excellent response/recovery time (22/22 ms), and stable fatigue cycle performance of up to 20,000 cycles under ambient humidity conditions. In recent years, Zhang et al. [44]. have studied flexible pressure sensors based on PDMS-carbon nanotubes. However, due to limitations in material sources and preparation processes, this research still has certain shortcomings in terms of sustainability. This study proposes a hydrogel film sensor based on biomass-derived CNPs and PVA composite films, combined with sandpaper templates, which has the advantages of being environmentally friendly, easy to prepare, and stable in performance.

**Table 1:** A comparison of this study with recent reports in the literature.

Sensing Material	Sensitivity (kPa^−1^)	Cyclic Durability	Response/Recovery Time (ms)	Ref.
Ionic-liquid/TPU complex	40.64 (0–1 kPa) 0.28 (1000–4000 kPa)	6000	41/58	[48]
Carbomer/rGo composite	3.1 (0–13 kPa) 0.22 (13–400 kPa)	10,000	14.9/22.4	[49]
Graphene coated micro-patterned PDMS	18.94 (0–40 kPa)	~5500	284/102	[50]
Interlink PDMS/CNT network	0.15 (0–47 kPa) 0.08 (47–214 kPa)	10,000	6/6	[44]
Patterned Ecoflex/rGO composite	4.68 (0–140 kPa) 11.09 (140–200 kPa)	1000	N/A	[51]
Sandpaper templated MXene/PDMS	3.94 (0–7.5 kPa) 0.0564 (27–119 kPa)	7500	70/84	[52]
Graphene coated PDMS	3.54 (0–100 kPa) 18.87 (100–140 kPa)	>10,000	125/129	[53]
ZnO/MXene/PU fibrous membrane	35.8 (0.2–35 kPa) 6.1(35–120 kPa) 1.2 (120–260 kPa)	10,000	100/60	[54]
Micro-structured PDMS coated with CNTs/ PPNWFs	6.31 (0–50 kPa) 3.07 (50–800 kPa)	10,000	72/88	[55]
Hemispherical graded micro-structured PDMS	1.357 (0–5 kPa) 0.077 (5–50 kPa)	6000	50/60	[56]
PEDOT: PSS coated PDMS	2.32 (0–100 kPa)	3000	240/100	[57]
***PVA/CNPs***	***101 (0–44.8 kPa)***	***20,000***	***22/22***	***This work***

### 2.3. Pressure Identification of PVA/CNPs Hydrogel Film Sensor

**Figure 3:**
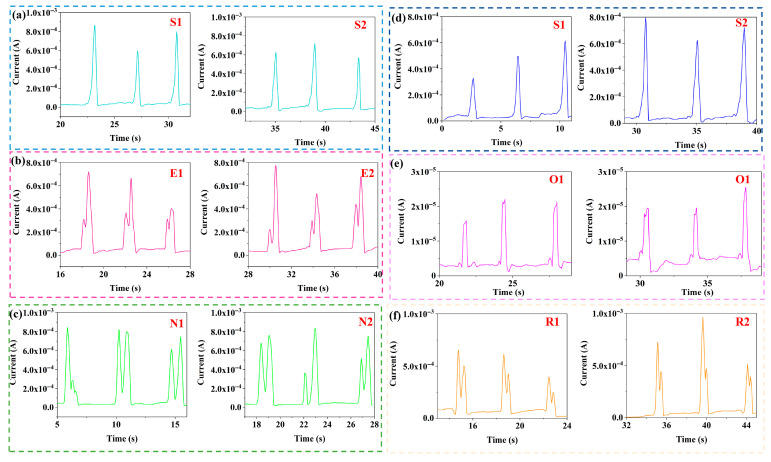
Current response signals generated by the PVA/CNPs composite hydrogel film sensor when writing different English letters using an ordinary signature pen: (**a**) “S”, (**b**) “E”, (**c**) “N”, (**d**) “S”, (**e**) “O”, and (**f**) “R”.

PVA/CNPs hydrogel film sensors have demonstrated excellent performance in a variety of real-world application scenarios. As shown in Figure 3, the letters “S”, “E”, “N”, “O” and “R” were entered and written twice each to test their stability. The results showed that identical letters produced consistent waveforms and current signals, while different letters corresponded to different waveform shapes and current amplitudes. This ability to generate unique current responses for different English characters highlights the potential of PVA/CNPs composite hydrogel sensors for handwriting recognition applications.

**Figure 4:**
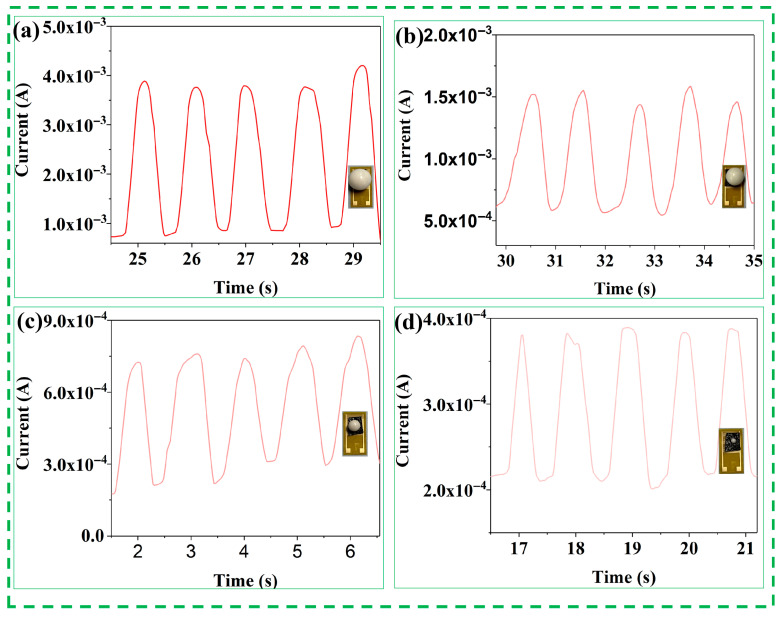
Current response signals of PVA/CNPs composite hydrogel film sensors under cyclic stress loading with different weights of spheres: (**a**) large spheres, (**b**) medium spheres, (**c**) small spheres, and (**d**) microspheres.

To further investigate the current signal response of the PVA/CNPs hydrogel film sensor to objects of different weights, current feedback tests were performed using four spherical objects of different weight classes (large, medium, small, and microspheres), as shown in Figure 4. The mass of these spheres varied to simulate different levels of pressure. Notably, the sensors showed significant differences in current values for different-sized spheres. Even microspheres of very small mass produce a perceptible current response. As the mass of the sphere increases, the relative strength of the electrical response increases.

**Figure 5:**
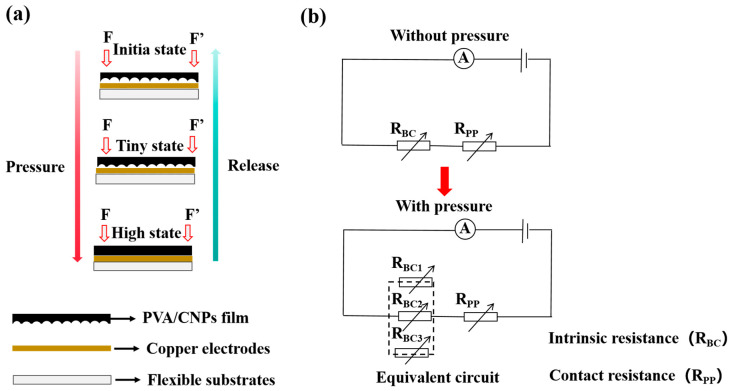
Working mechanism of PVA/CNPs hydrogel film sensor. (**a**) Simulation diagram of the film under applied and unapplied force conditions. (**b**) Representation of the associated equivalent circuit model.

In order to reveal more comprehensively the sensing mechanism of the PVA/CNPs hydrogel film sensor and its response characteristics to external pressure, its operating principle is shown in Figure 5. Figure 5a shows the schematic structure of the sensor under applied pressure, and Figure 5b corresponds to its equivalent circuit model. The total resistance of the conductive path of the thin-film sensor consists of two main components: the intrinsic resistance of the hydrogel film body (R_PP_), and the interfacial contact resistance (R_BC_) between the bottom of the film and the copper foil electrode. As shown in Figure 5a, in the initial state without applying an external force, the surface of the PVA/CNPs hydrogel film sensor with sandpaper microstructure on one side could not be completely adhered to the copper foil electrode, and only the higher raised portion of the surface formed a contact with the electrode, which constituted a limited conductive pathway, and at this time, the interfacial contact resistance (R_BC_) was larger. It is well known that the total resistance of the parallel pathway decreases with the increase in the number of parallel branches. When external pressure is applied, the sandpaper-like microstructure on the surface of the film deforms, and more raised areas contact the copper foil electrode, which significantly increases the contact area and the number of contact points, and the number of conductive paths increases accordingly, and the interfacial resistance, R_BC_, decreases significantly as a result (see Figure 5b). When the applied pressure reaches a certain level, the sandpaper-like microstructure on the film surface is completely compressed and deformed, enabling full contact between the hydrogel film and the copper foil electrode. At this time, the interface is equivalent to a large number of parallel contact resistors, the electron transport path is significantly shortened [38], and the R_BC_ is further reduced, which contributes to the continuous change in the total device resistance, and thus enhances the sensitivity and pressure response range of the PVA/CNPs hydrogel film sensor.

### 2.4. Applications for Human Activity Monitoring

**Figure 6:**
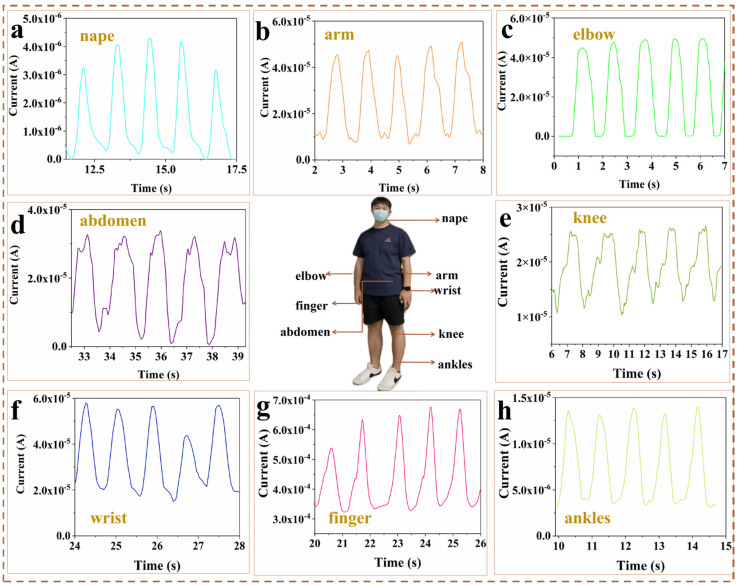
Practical applications for human motions and physiological signals monitoring. (**a**) Changes in electrical signals at the neck after nodding the head. (**b**) Changes in electrical signals when bending the elbow intra-articularly. (**c**) Changes in electrical signals during external elbow flexion. (**d**) Current responses corresponding to abdominal breathing exercises. (**e**) Current changes during knee flexion. (**f**) Monitoring the electronic response to wrist flexion. (**g**) The change in current when bending the index finger. (**h**) Current changes during ankle flexion.

The flexible strain sensors with a sandpaper-like microstructure offer high sensitivity, low detection limits, wide sensing ranges, fast response times, and excellent stability and durability, making them ideal for monitoring human movements and physiological signals. As shown in Figure 6, the sensors were attached to different parts of the human body to fully demonstrate their prospects for wide application as flexible wearable devices. When the sensor is mounted on the back side of the neck (Figure 6a), the movement status of the neck can be accurately monitored. The greater the bending angle, the greater the tensile deformation of the sensor, which leads to a greater change in resistance, which allows for an accurate quantification of the degree of movement of the neck. When the sensor is attached to the inner arm muscles (Figure 6b), the sensor is deformed by the bending of the arm during a simple extension and retraction arm movement, resulting in a real-time change in relative resistance. When the sensor is attached to the elbow joint of the body (Figure 6c), a stable electrical response signal is recorded during everyday activities such as joint flexion. During the cycle of exhalation and inhalation, there are corresponding concave and convex movements of the abdomen that put pressure on the sensor, resulting in a change in resistance that allows monitoring of the respiratory state (Figure 6d). Knee function is an important indicator for monitoring patients with certain orthopaedic conditions, and when the sensor is attached to a person’s knee joint, the sensor captures real-time electrical signals as the knee joint moves (Figure 6e). The sensor mounted on the wrist (Figure 6f) accurately monitors the flexion and extension of the wrist, and a corresponding change in current can be observed when the wrist joint is repeatedly flexed. When attached to the finger (Figure 6g), it accurately detects finger flexion and extension. When attached to the ankle (Figure 6h), it accurately detects the dorsiflexion and extension states, which is particularly important for activities involving balance and movement (e.g., dancing or exercising). The experimental results show that the sensors developed in this study can effectively detect various movement states and physiological signals of the human body with high accuracy. In addition, these sensors have excellent flexibility and an ultra-thin design that fits seamlessly onto human skin or clothing, making them ideal for applications such as electronic skin, human–machine interfaces, and soft robotics.

### 2.5. Deep Learning Analysis of Breathing Phase Classification Using a Single PVA/CNPs Hydrogel Film Sensor

Abdominal monitoring has unique advantages in health assessment due to its low environmental interference and non-invasive convenience. Deep learning techniques have been used to achieve high-precision gesture recognition of pressure sensor data (e.g., Thien Trung Luu et al. [58]), which provides a new way to improve the accuracy of data analysis of physiological signals such as abdominal breathing.

**Figure 7:**
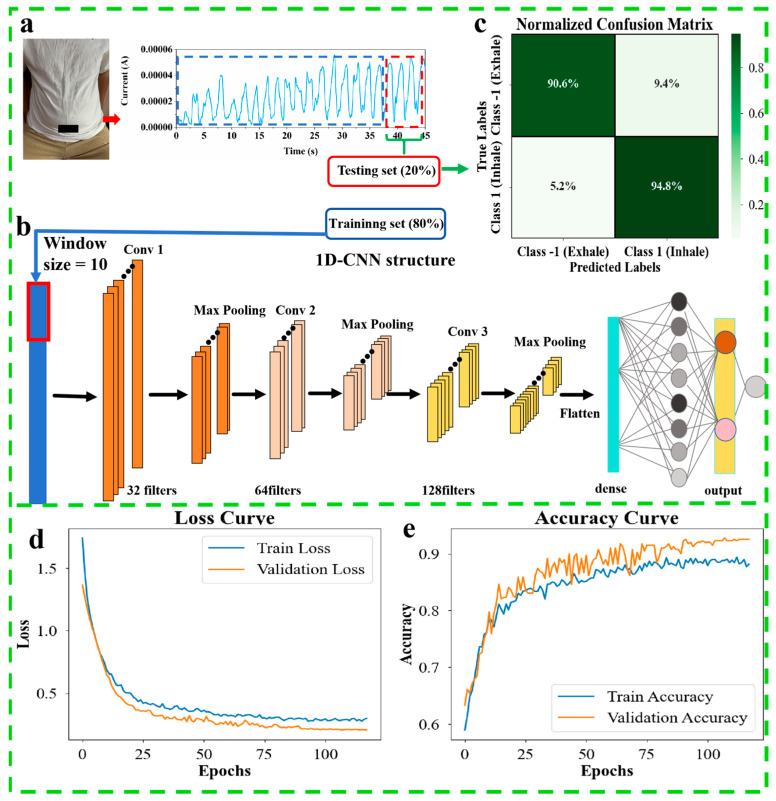
Respiratory phase classification using a single pressure sensor. (**a**): collection and segmentation of abdominal data, dividing the dataset into 80% training and 20% testing. (**b**): 1D CNN architecture with convolutional, pooling and dense layers for feature extraction and classification. (**c**): Confusion matrix showing classification accuracy for exhale (−1) and inhale (1). (**d**): Loss curves for more than 100 trainings and validations. (**e**): Accuracy curve for more than 100 training and validation.

In the experiments, the abdominal respiratory current signal and time data were acquired by a single PVA/CNPs hydrogel film sensor, which was first preprocessed and later labelled as exhale (−1) and inhale (1) phases, generating a total of 1967 data points. After completion of the dataset building, the preprocessed data were directly fed into a 1D CNN model for classification (Figure 7a: 80% training, 20% testing). The model structure consists of three convolutional layers (32/64/128 filters), a pooling layer and a dense layer, and the input signal is processed with a window size of 10 segments (Figure 7b). 1D-CNN model batch size: 32, optimiser: Adam, learning rate: 0.001, epoch: 500. Dropout, batch normalisation, early stopping, and learning rate adaptation strategies were used to reduce the risk of overfitting. The results show that the accuracy of the normalised confusion matrix is 90.6% for exhale recognition and 94.8% for inhale recognition (Figure 7c); the loss curve smoothly decreases to the low-value region during the training process, and the accuracy quickly converges to a high level (Figure 7d,e), which verifies the robustness of the model in classifying respiratory signals. Accurate classification of breathing phases provides a new method for monitoring human respiratory health and contributes to the application of wearable sensors in the medical field.

## 3. Conclusions

In this study, we prepared a flexible PVA/CNPs hydrogel film sensor with a sandpaper-derived microstructure. The sensor exhibited excellent sensitivity (101 kPa^−1^), fast response/recovery time (22 ms). Applications for the sensor include letter recognition, motion capture and respiratory monitoring: by detecting the abdominal respiratory current signal and combining it with a 1D-CNN, the sensor classifies respiratory phases with more than 90 per cent accuracy, providing a new solution for non-invasive monitoring of human respiratory health. After undergoing 20,000 fatigue cycles, the performance of the sensor still declines to some extent due to the limitations of the sandpaper template structure. Future research needs to further optimise the structural design to overcome this limitation. At the same time, such sensors have broad application potential in clinical monitoring and robot interaction fields, fully demonstrating the broad prospects for material engineering and artificial intelligence to work together to solve wearable technology challenges.

## 4. Materials and Methods

### 4.1. Materials

Polyvinyl Alcohol 1750 ± 50 was purchased from Sinopharm Chemical Reagent Co., Ltd. (Chengdu, China). Cuttlefish ink was purchased from Xinjian Aquatic Company, Qingdao, China.

### 4.2. Preparation of CNPs

The CI was immersed in deionized water at room temperature overnight, followed by centrifugation at 2000 r/min for 5 min to remove larger particles. This was repeated at 9000 r/min for 10 min, five times, to eliminate impurities (Shanghai Lu Xiangyi Centrifuge Instrument Co., Ltd., Shanghai, China). The resulting precipitate was dried at 50 °C for 24 h and ground for 10 min. The material was then placed in quartz boats and heated in a tube furnace (Hefei Kejing Materials Technology Co., Ltd., Hefei, China) at a rate of 5 °C/min, holding at 900 °C for 2 h in an argon atmosphere to synthesise CNPs.

### 4.3. Fabrication of PVA/CNPs Hydrogel Film Sensor

**Figure 8:**
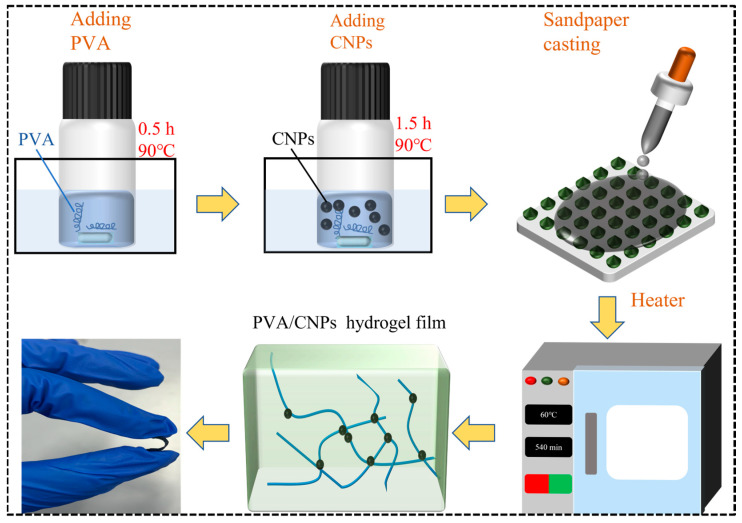
Schematic diagram of the preparation process of PVA/CNPs hydrogel film sensor. CNPs were placed into a fully dissolved PVA solution and mixed with magnetic stirring to form a homogeneous PVA/CNPs solution. The solution was then cast and solidified on sandpaper to form a PVA/CNPs hydrogel film sensor with a sandpaper microstructure.

Figure 8 shows a schematic diagram of the preparation of PVA/CNPs hydrogel film sensor. In order to investigate the effect of different concentrations of CNPs on the sensor performance of the composite films, two different PVA/CNPs hydrogel film sensor with different ratios of PVA to CNPs were prepared: 10:1 and 10:2. For the specific experimental procedure, 3 g of PVA was dissolved in deionised water and magnetically stirred at 90 °C for 2 h. In addition, 0.3 g and 0.6 g of CNPs were dispersed in deionised water and treated with ultrasonic waves (Kunshan Ultrasonic Instrument Co., Ltd., Kunshan, China). In addition, 0.3 g and 0.6 g of CNPs were dispersed in deionised water and ultrasonicated for 30 min to ensure homogeneous dispersion. These dispersions were labelled as PVA/CNPs-1 (10:1) and PVA/CNPs-2 (10:2), respectively. Each dispersion was mixed into a PVA solution and ultrasonicated for 30 min. The resulting mixture solution was poured onto a 100 mesh sandpaper template and dried at 70 °C for 6 h to form a composite film with a textured surface.

### 4.4. CNPs Morphology Characterisation

The morphology of CNPs was examined using a ZEISS Gemini Sigma 300 scanning electron microscope (SEM) (Zeiss, Oberkochen, Germany), revealing predominantly spherical particles.

### 4.5. Characterisation

Surface morphology of CNPs and PVA/CNPs composite films was analysed using a ZEISS Gemini Sigma 300 SEM. FT-IR spectra were recorded on a SHIMADZU spectrophotometer (SHIMADZU, Tokyo, Japan). The graphitization degree was assessed using a Renishaw inVia Raman microscope with a 532 nm laser (Thermo Fisher Scientific Inc., Waltham, MA, USA). Stable pressure was applied to the sensors using an Eidelberg HLD Spiral Machine Stress Tester (Yueqing, China), and the cyclic stress test was conducted at a load pressure of 1 kPa, a frequency of 2 Hz, and room temperature using equipment from ShengDa Machinery (ShengDaMachinery, Tianjin, China). Electrical signals were monitored using NS-SourceMeter software and a Keithley 2602 source metre (Manufactured by Tektronix, Cleveland, OH, USA).

### 4.6. 1D-CNN Model

1D-CNN can more effectively identify complex structures and patterns in input data through its automated feature extraction mechanism, thereby improving model performance. This method has been applied to one-dimensional time series analysis and can adapt to data inputs of different lengths and shapes, making it highly practical for various signal processing tasks [59].

## Data Availability

The original contributions presented in this study are included in the article. Further inquiries can be directed to the corresponding author.

## Abbreviations

The following abbreviations are used in this manuscript:

PVA	polyvinyl alcohol
1D-CNN	one-dimensional convolutional neural networks
CNPs	carbon nanospheres
CI	cuttlefish ink

## References

[1] Stoppa M., Chiolerio A. (2014). Wearable electronics and smart textiles: A critical review. Sensors.

[2] Sekine T., Abe M., Muraki K., Tachibana S., Wang Y.F., Hong J., Tokito S. (2020). Microporous induced fully printed pressure sensor for wearable soft robotics machine interfaces. Adv. Intell. Syst..

[3] Rangarajan S.l, Kidane A., Qian G., Rajko S., Birchfield D. (2007). The design of a pressure sensing floor for movement-based human computer interaction. Smart Sensing and Context.

[4] Kim S., Amjadi M., Lee T.I., Jeong Y., Kwon D., Kim M.S., Park I. (2019). Wearable, ultrawide-range, and bending-insensitive pressure sensor based on carbon nanotube network-coated porous elastomer sponges for human interface and healthcare devices. ACS Appl. Mater. Interfaces.

[5] Annese V.F., De Venuto D., Martin C., Cumming D.R. Biodegradable pressure sensor for health-care. Proceedings of the 21st IEEE International Conference on Electronics, Circuits and Systems (ICECS).

[6] Pierre Claver U., Zhao G. (2021). Recent progress in flexible pressure sensors based electronic skin. Adv. Eng. Mater. Adv. Eng. Mater..

[7] Nguyen T., Dinh T., Phan H.P., Pham T.A., Dau V.T., Nguyen N.T., Dao D.V. (2021). Advances in ultrasensitive piezoresistive sensors: From conventional to flexible and stretchable applications. Mater. Horiz..

[8] Liu C., Xu Z., Chandrasekaran S., Liu Y., Wu M. (2023). Self-healing, antibacterial, and conductive double network hydrogel for strain sensors. Carbohydr. Polym..

[9] Gu J., Huang J., Chen G., Hou L., Zhang J., Zhang X., Liu H. (2020). Multifunctional poly (vinyl alcohol) nanocomposite organohydrogel for flexible strain and temperature sensor. ACS Appl. Mater. Interfaces.

[10] Zhu B., Niu Z., Wang H., Leow W.R., Li Y., Zheng L., Chen X. (2014). Microstructured graphene arrays for highly sensitive flexible tactile sensors. Small.

[11] Wang Q., Ling S., Liang X., Wang H., Lu H., Zhang Y. (2019). Self-healable multifunctional electronic tattoos based on silk and graphene. Adv. Funct. Mater..

[12] Cheng R., Zeng J., Wang B., Li J., Cheng Z., Xu J., Chen K. (2021). Ultralight, flexible and conductive silver nanowire/nanofibrillated cellulose aerogel for multifunctional strain sensor. Chem. Eng. J..

[13] Yang Y., Sun N., Wen Z., Cheng P., Zheng H., Shao H., Lee S.T. (2018). Liquid-metal-based super-stretchable and structure-designable triboelectric nanogenerator for wearable electronics. ACS Nano.

[14] Chen T., Yang G., Li Y., Li Z., Ma L., Yang S., Wang J. (2022). Temperature-adaptable pressure sensors based on MXene-coated GO hierarchical aerogels with superb detection capability. Carbon.

[15] Han F., Luo J., Pan R., Wu J., Guo J., Wang Y., Zhang Q. (2022). Vanadium dioxide nanosheets supported on carbonized cotton fabric as bifunctional textiles for flexible pressure sensors and zinc-ion batteries. ACS Appl. Mater. Interfaces.

[16] Long W., Yao Y., Ye Y., Zhang C., Xu J., Zhong D., Xia L. (2025). Flexible strain sensor based on carbonized corn stalk with three-dimensional network. Ind. Crops Prod..

[17] Karthikeyan N., Vijayalakhmi K.A. (2025). Low temperature plasma exposed activated peanut shell carbon with Al-doped CdO air cathode is employed in Al-air batteries and supercapacitors. J. Power Sources.

[18] Heydari M., Moheb A., Ghiaci M., Masoomi M. (2013). Effect of cross-linking time on the thermal and mechanical properties and pervaporation performance of poly(vinyl alcohol) membrane cross-linked with fumaric acid used for dehydration of isopropanol. J. Appl. Polym. Sci..

[19] Hashim H., Abdallh M., Yousif E. (2012). Studying the influence of cobalt chloride on the optical properties of poly (vinyl alcohol) films. J. Al-Nahrain Univ..

[20] Fernandes D.M., Andrade J.L., Lima M.K., Silva M.F., Andrade L.H.C., Lima S.M., Pineda E.G. (2013). Thermal and photochemical effects on the structure, morphology, thermal and optical properties of PVA/Ni_0.04_Zn_0.96_O and PVA/Fe_0.03_Zn_0.97_O nanocomposite films. Polym. Degr. Stab..

[21] Sreeja S., Sreedhanya S., Smijesh N., Philip R., Muneera C.I. (2013). Organic dye impregnated poly(vinyl alcohol) nanocomposite as an efficient optical limiter: Structure, morphology and photophysical properties. J. Mater. Chem. C.

[22] Sreedhar S., Illyaskutty N., Sreedhanya S., Philip R., Muneera C.I. (2016). An organic dyepolymer (phenol red-poly(vinyl alcohol)) composite architecture towards tunableoptical and -saturable absorption characteristics. J. Appl. Phys..

[23] Kim Y.R., Kim M.P., Park J., Lee Y., Ghosh S.K., Kim J., Ko H. (2020). Binary Spiky/spherical nanoparticle films with hierarchical micro/nanostructures for high-performance flexible pressure sensors. ACS Appl. Mater. Interfaces.

[24] Tee B.C.K., Chortos A., Dunn R.R., Schwartz G., Eason E., Bao Z. (2014). Tunable flexible pressure sensors using microstructured elastomer geometries for intuitive electronics. Adv. Funct. Mater..

[25] Li T., Luo H., Qin L., Wang X., Xiong Z., Ding H., Zhang T. (2016). Flexible capacitive tactile sensor based on micropatterned dielectric layer. Small.

[26] Xiao Y., Hu C., Yang L., Wu J., Li J. (2025). Preparation of graphene/polydimethylsiloxane flexible resistive pressure sensors based on direct ink writing 3D printing. Sens. Actuators A Phys..

[27] Zhang X., Lu L., Wang W., Zhao N., He P., Liu J., Yang B. (2022). Flexible pressure sensors with combined spraying and self-diffusion of carbon nanotubes. ACS Appl. Mater. Interfaces.

[28] Song K., Zhao R., Wang Z.L., Yang Y. (2019). Conjuncted pyro-piezoelectric effect for self-powered simultaneous temperature and pressure sensing. Adv. Mater..

[29] Guo H., Pu X., Chen J., Meng Y., Yeh M.H., Liu G., Wang Z.L. (2018). A highly sensitive, self-powered triboelectric auditory sensor for social robotics and hearing aids. Sci. Robot..

[30] He Q., Wu Y., Feng Z., Sun C., Fan W., Zhou Z., Yang J. (2019). Triboelectric vibration sensor for a human-machine interface built on ubiquitous surfaces. Nano Energy.

[31] Zhu M., Sun Z., Zhang Z., Shi Q., He T., Liu H., Lee C. (2020). Haptic-feedback smart glove as a creative human-machine interface (HMI) for virtual/augmented reality applications. Sci. Adv..

[32] Wang J., Chen Y., Hao S., Peng X., Hu L. (2019). Deep learning for sensor-based activity recognition: A survey. Pattern Recognit. Lett..

[33] LeCun Y., Bengio Y., Hinton G. (2015). Deep learning. Nature.

[34] Zhu M., He T., Lee C. (2020). Technologies toward next generation human machine interfaces: From machine learning enhanced tactile sensing to neuromorphic sensory systems. Appl. Phys. Rev..

[35] Ahmadzadeh M., Zahrai S.M., Bitaraf M. (2025). An integrated deep neural network model combining 1D CNN and LSTM for structural health monitoring utilizing multisensor time-series data, Struct. Health Monit..

[36] Shi Q., Zhang Z., He T., Sun Z., Wang B., Feng Y., Lee C. (2020). Deep learning enabled smart mats as a scalable floor monitoring system. Nat. Commun..

[37] Chen K., Wang W., Ye Z., Dong Y., Wan L., Zhang Z., Wang Z. (2024). In Situ graft-on fibrous composites and nanostructure interlocking facilitate highly stable wearable sensors for sids prevention. Adv. Fiber Mater..

[38] Ni Y., Liu L., Huang J., Li S., Chen Z., Zhang W., Lai Y. (2022). Rational designedmicrostructure pressure sensors with highly sensitive and wide detection range performance. J. Mater. Sci. Technol..

[39] Bai N., Wang L., Wang Q., Deng J., Wang Y., Lu P., Guo C.F. (2020). Graded intrafillable architecture-based iontronic pressure sensor with ultra-broad-range high sensitivity. Nat. Commun..

[40] Taspika M., Permatasari F.A., Nuryadin B.W., Mayangsari T.R., Aimon A.H., Iskandar F. (2019). Simultaneous ultraviolet and first near-infrared window absorption of luminescent carbon dots/PVA composite film. RSC Adv..

[41] Yang W., Han X., Yin C., Zhang X., Peng Q., Yi C. (2024). Biomass carbon nanosphere-based piezoresistive flexible pressure sensors for motion capture and health monitoring. Compos. Commun..

[42] Nešović K., Janković A., Kojić V., Vukašinović-Sekulić M., Perić-Grujić A., Rhee K.Y., Mišković-Stanković V. (2018). Silver/poly(vinyl alcohol)/chitosan/graphene hydrogels synthesis, biological and physicochemical properties and silver release kinetics. Compos. Part B Eng..

[43] Pang H., Xu L., Yan D.X., Li Z.M. (2014). Conductive polymer composites with segregated structures. Prog. Polym. Sci..

[44] Zhang Y., Yang J., Hou X., Li G., Wang L., Bai N., Guo C.F. (2022). Highly stable flexible pressure sensors with a quasi-homogeneous composition and interlinked interfaces. Nat. Commun..

[45] Zheng W., Yang Y., Fan L., Ye D., Xu W., Xu J. (2023). Ultralight PPy@PVA/BC/MXene composite aerogels for high-performance, supercapacitor eltrodes and pressure sensors. Appl. Surf. Sci..

[46] Luo F., Chen B., Ran X., Ouyang W., Yao Y., Shang L. (2023). Wearable and self-powered triboelectric sensors based on NaCl/PVA hydrogel for driver multidimensional information monitoring. Nano Energy.

[47] Chen Z., Ma Y., Wang H., Yu B., Qian L., Zhao Z.J. (2024). Starfish-inspired ultrasensitive piezoresistive pressure sensor with an ultra-wide detection range for healthcare and intelligent production. Chem. Eng. J..

[48] Hyun J.E., Lim T., Kim S.H., Lee J.H. (2024). Wearable ion gel based pressure sensor with high sensitivity and ultra-wide sensing range for human motion detection. Chem. Eng. J..

[49] Cao K., Wu M., Bai J., Wen Z., Zhang J., Wang T., Jiang L. (2022). Beyond skin pressure sensing: 3D printed laminated graphene pressure sensing material combines extremely low detection limits with wide detection range. Adv. Funct. Mater..

[50] Zhang Y., Lu Q., He J., Huo Z., Zhou R., Han X., Zhai J. (2023). Localizing strain via micro-cage structure for stretchable pressure sensor arrays with ultralow spatial crosstalk. Nat. Commun..

[51] Wu Q., Qiao Y., Guo R., Naveed S., Hirtz T., Li X., Ren T.L. (2020). Triode-mimicking graphene pressure sensor with positive resistance variation for physiology and motion monitoring. ACS Nano.

[52] Jia B., Li Z., Zheng T., Wang J., Zhao Z.J., Zhao L., Jiang Z. (2024). Highly-sensitive, broad-range, and highly-dynamic MXene pressure sensors with multi-level nano-microstructures for healthcare and soft robots applications. Chem. Eng. J..

[53] Meng X., Zhang C., Xie H., Niu S., Han Z., Ren L. (2024). A continuous pressure positioning sensor with flexible multilayer structures based on a combinatorial bionic strategy. Adv. Funct. Mater..

[54] Lei P., Bao Y., Zhang W., Gao L., Zhu X., Xu J., Ma J. (2024). Synergy of ZnO nanowire arrays and electrospun membrane gradient wrinkles in piezoresistive materials for wide-sensing range and high-sensitivity flexible pressure sensor. Adv. Fiber Mater..

[55] Cui X., Jiang Y., Hu L., Cao M., Xie H., Zhang X., Zhu Y. (2023). Synergistically microstructured flexible pressure sensors with high sensitivity and ultrawide linear range for full-range human physiological monitoring. Adv. Mater. Technol..

[56] Su S., Zhang X., Dang D., Wang Z., Tong Z. (2024). A high-performance flexible capacitive pressure sensor with 3-d printed hemispherical graded microstructures. IEEE Sens. J..

[57] Zhang H., Chen X., Liu Y., Yang C., Liu W., Qi M., Zhang D. (2024). PDMS film-based flexible pressure sensor array with surface protruding structure for human motion detection and wrist posture recognition. ACS Appl. Mater. Interfaces.

[58] Luu T.T., Le H.A.T., Ra Y., Weldemhret T.G., Kim H., Choi K., Park Y.T. (2025). Recovered graphene-hydrogel nanocomposites for multi-modal human. Compos. Part B Eng..

[59] Zhao X., Wen Z., Pan X., Ye W., Bermak A. (2019). Mixture gases classification based on multi-label one-dimensional deep convolutional neural network. IEEE Access.

